# An Easier Technique for End to End Pancreatlcojejunostomy

**DOI:** 10.1155/1996/18087

**Published:** 1996

**Authors:** Sergio Pedrazzoli, Cosimo Sperti, Claudio Pasquali

**Affiliations:** 1 Department of Semeiotica Chirurgica University of Padua Italy

## Abstract

Breakdown of the pancreaticoenterostomy is responsible for a number of complications and for the high mortality associated with pancreaticoduodenectomy. Although in recent years the postoperative mortality has dropped to less than 10% and in some to less than 5%, pancreatic fistula remains the most common and troublesome complication.

Various procedures, such as duct ligation or occlusion, resection of the pancreatic stump or pancreaticogastrostomy, have been proposed to treat the pancreatic stump when it is considered unsuitable for jejunal anastomosis. A little trick permitted us to perform 41 consecutive end to end pancreaticojejunostomies, irrespective of the conditions of the pancreatic stump, with only 3 pancreatic fistulas (7%) and without fistula related deaths.


*HPB Surgery*, 1996, Vol. 9, pp.141–143
Reprints available directly from the publisher
Photocopying permitted by license only

© 1996 OPA (Overseas Publishers Association)
Amsterdam B.V. Published in The Netherlands
by Harwood Academic Publishers GmbH
Printed in Malaysia

### An Easier Technique for End to End Pancreaticojejunostomy

SERGIO PEDRAZZOLI, COSIMO SPERTI and CLAUDIO PASQUALI

Department of Semeiotica Chirurgica, University of Padua, Italy

*(Received 28 October 1993)*

Breakdown of the pancreaticoenterostomy is responsible for a number of complications and for the high mortality associated with pancreaticoduodenectomy. Although in recent years the postoperative mortality has dropped to less than 10% and in some to less than 5%, pancreatic fistula remains the most common and troublesome complication.

Various procedures, such as duct ligation or occlusion, resection of the pancreatic stump or pancreaticogastrostomy, have been proposed to treat the pancreatic stump when it is considered unsuitable for jejunal anastomosis. A little trick permitted us to perform 41 consecutive end to end pancreaticojejunostomies, irrespective of the conditions of the pancreatic stump, with only 3 pancreatic fistulas (7 %) and without fistula related deaths.

KEY WORDS: Pancreas pancreatic surgery pancreaticojejunostomy pancreatic anastomosis

### INTRODUCTION

Pancreaticoduodenectomy has been the standard operation for tumors of the pancreatic head. Although in recent years the postoperative mortality rate has dropped to less than 10% and in some to less than 5% (1), pancreatic fistula remains the most common and troublesome complication (2). "Normal" pancreas is the most difficult organ for an anastomosis. We present here a little trick to perform an easy end to end pancreaticojejunostomy whatever the condition of the pancreatic stump.

### PATIENTS AND METHODS

Pancreaticoduodenectomy was performed in 39 consecutive patients from September 1986 to February 1993; two further insulinoma patients underwent duodenum preserving resection of the head of the pancreas (Tab. 1) with pancreaticojejunostomy of the remnant body-tail of the pancreas. No pancreatic stump or duct were considered unsuitable for anastomosis, as follows:

**Table I** Patient characteristics

| *Background factors* | *n = 41* |
|---|---|
| Age, y | 62.5 ± 11.2 |
| Sex, N°M/F | 25 / 16 |
| Obstructive jaundice, N. (%) | 29 (71) |
| Diabetes mellitus, N. (%) | 10 (24) |
| Pancreatic tumor @, N. (%) | 24 (59) |
| Endocrine tumor, * N. (%) | 3 (7) |
| Chronic pancreatitis, N. (%) | 5 (12) |
| Ampullary tumors, N. (%) | 7 (17) |
| Choledocho duodenal tumors N. (%) | 2 (5) |
| MPD dilatation, # N. (%) | 11 (27) |
| Fibrotic pancreas **, N. (%) | 11 (34) |
| Normal pancreas, N. (%) | 22 (54) ## |

@: Pancreatic cancer, 23 cases; pancreatic cystoadenocarcinoma, 1 case.
*: Two duodenum-preserving pancreas head resection.
#: The main pancreatic duct (MPD) was 4 mm or more in diameter.
**: Severe acinar cell atrophy with prominent fibrosis.
##: Three pancreatic fistulas (13.6%).

*Correspondence and reprint request to:* Sergio Pedrazzoli MD, FACS, Direttore Institut di SemeioticaChirurgica, Università di Padova, Via Facciolati, 71, 35127 PADOVA, ITALY, Phone: 039/49/8216735 Fax: 039/49/8021066

After completion of resection, the pancreatic stump is freed from the surrounding vessels and structures for a length of about 2.5 cm (Fig. 1). A jejunal loop is prepared for the pancreatico-jejunal anastomosis by removing the seromuscular layer of the first 2–3 cm of the bowel where it will be anastomosed (Fig. 2). A first layer of interrupted three zeros slow-absorbable material is created between the posterior surface of the pancreas about 2 cm from transection line and the seromuscular layer of the bowel (Fig. 3). The mucosal edge is then sutured by a continuous layer of the same material to the edge of the pancreatic stump. No stent is inserted in the pancreatic duct. An anterior layer of interrupted three zeros slow absorbing material suture is now applied between the anterior seromuscular border of the bowel and the anterior surface of the pancreatic stump. By means of this layer, the first 2 cm of pancreatic stump are progressively invaginated in to the bowel (Fig. 4).

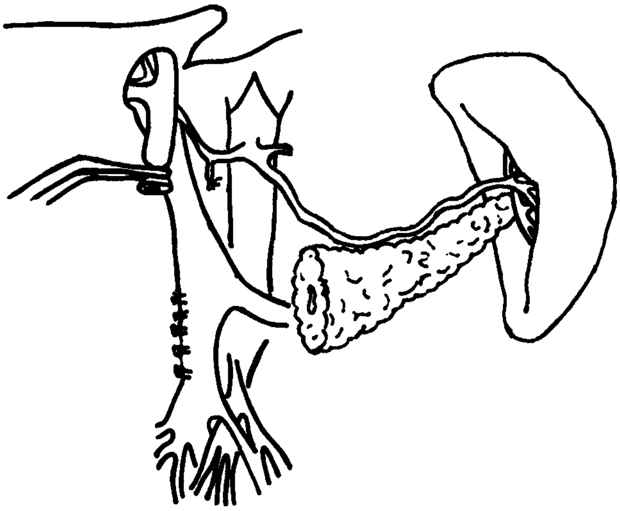

**Figure 1** The body of the pancreas is transected and freed from the surrounding vessels and structures for a length of 3 cm (1.2 in).

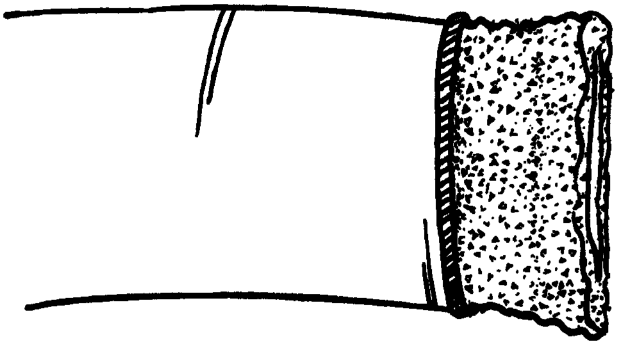

**Figure 2** Jejunum is deprived of seromuscular layer on its last 2 cm (0.8 in).

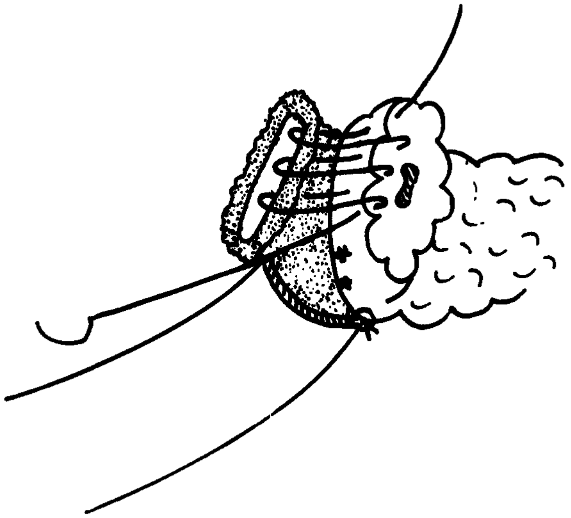

**Figure 3** A posterior layer of interrupted three-zero Dexon suture is prepared. One layer running three-zero Dexon between the free mucosal border and the border of pancreatic stump is carried out.

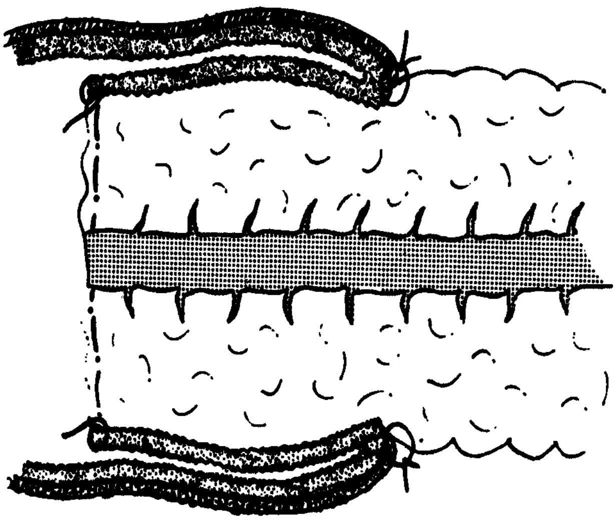

**Figure 4** Pancreatic stump is invaginated with interupted three-zero Dexon between pancreatic capsule and the anterior seromuscular layer of jejunum.

### RESULTS

Pancreaticoduodenectomy was performed in 39 consecutive patients from September 1986 to February

1993; two further insulinoma patients underwent duodenum preserving resection of the head of the pancreas (Tab. 1) with pancreaticojejunostomy of the remnant body-tail of the pancreas. Pancreaticojejunostomy was therefore performed in 41 patients.

Three patients died after surgery: the first one died two days after surgery of peritoneal haemorrhage from the sutured splenic artery and subsequent DIC; this patient was not considered in the results. The second died 16 days after surgery of faecal peritonities from a perforated colonic diverticulum. The third patient had a stormy postoperative course, and died three months after surgery, because, of extraabdominal sepsis. At post mortem examination in the last 2 patients the pancreaticojejunal anastomosis was intact.

Postoperatively, two patients showed late, minor pancreatic fistulas and another a late major fistula. All closed spontaneously with conservative treatment. No fistula was observed in patients with pancreatic duct dilatation and/or fibrotic pancreas (Table 1).

### DISCUSSION

Despite reduction of operative mortality for pancreaticoduodenectomy, intraabdominal complications follow in 25% of patients and anastomotic leaks remain a primary concern (2-4). Leakage rate of pancreaticojejunostomy after pancreaticoduodenectomy varies from 0 (4) to 60% (5) with a mean rate of 13% (2) and a mortality rate in fistula patients of about 20% (2). Furthermore many Surgeons considered in this review have suggested total pancreatectomy or duct occlusion when remnant pancreatic stump was considered unfit for anastomosis. Although we did not consider any patient unsuited for anastomosis, and dilatation of the main pancreatic duct and/or a fibrotic pancreas were observed in only one third of our cases, only three fistulas (7%) have been observed among 40 patients (the patient who died two days after surgery was excluded); the percentage found lies within the lower limits of incidence reported in the literature (2). Furthermore no pancreatitis of the remnant pancreas has been observed. End to end pancreatico-jejunal anastomosis is preferred by most pancreatic surgeons (2). Although our results are not so different from that reported by Griffanti Bartoli's review (2), our invagination procedure may be easier than that generally in use and will allow us to perform an end to end anastomosis even with an enlarged, soft pancreas. Furthermore, because of this little trick, a single muscular layer (instead of two layers created with the usual invagination procedure) envelops the pancreatic stump, lowering the risk of stump pancreatities. The short additional work added by denuding of seromuscolar layer of the jejunum is compensated by the quicker and easier performance of the end to end invagination of the pancreas.

Pancreatico-gastrostomy has also been proposed to prevent fistula formation (7). Only 1 fistula in 75 cases has been reported (2). Overall mortality is however the same.

### SUMMARY

Invagination technique of pancreatico-jejunostomy can be performed with relative simplicity, irrespective of pancreatic stump conditions, if interal cylinder of the jejunum is deprived of its seromuscular layer. In 41 consecutive anastomoses only three, late, pancreatic fistulas (7.3%) were observed. All closed spontaneously and no anastomosis-related death was observed.

### ACKNOWLEDGMENT:

This study was supported by grant 94.01179.PF39 from the Italian Research Council.

### REFERENCES

1. Grace, P.A., Pitt, H.A., Tompkins, R.K., DenBesten, L., Longmire, W.P. (1986) Decreased morbidity and mortality after pancreatoduodenectomy. *Am J Surg*, **151**, 141–149.
2. Griffanti Bartoli, F., Arnone, G.B., Ravera, G., Bachi, V. (1991) Pancreatic fistula and relative mortality in malignant disease after pancreaticoduodenectomy. Review and statistical metaanalysis regarding 15 years of literature. Anticancer Res, **11**, 1831–1848.
3. Papachristou, D.N., Fortner, J.G. (1981) Management of the pancreatic remnant in pancreatoduodenectomy. *J Surg Oncol*, **18**, 1–7.
4. Bittner, R., Roscher, R., Safi, F., Dopfer, H.P., Scholzel, E., Beger, H.G. (1989) Der einflub von tumorgrosse und lymphknotenstatus auf die prognose des pancreas-carcinoms. Chirurg, **60**, 240–245.
5. Balasegaram, M. (1976) Carcinoma of the periampullary region: a review of a personal series of 87 patients. *Br J Surg*, **63**, 532–537.
6. Brodsky, J.T., Turnbull, A.D.M. (1991) Arterial hemorrhage after pancreatoduodenectomy. The sentinel bled. *Arch Surg*, **126**, 1037–1040.
7. Obertop, H., van Houten, H. (1984) A new technique for pancreatojejunostomy. Surg Gynecol Obstet, **159**, 89–91.
8. Bradbeer, J.W., Johnson, C.D. (1990) Pancreaticogastrostomy after pancreaticoduodenectomy. *Ann R Coll Surg Engl*, **72**, 266–269.

